# Research on Non-Destructive Quality Detection of Sunflower Seeds Based on Terahertz Imaging Technology

**DOI:** 10.3390/foods13172830

**Published:** 2024-09-06

**Authors:** Hongyi Ge, Chunyan Guo, Yuying Jiang, Yuan Zhang, Wenhui Zhou, Heng Wang

**Affiliations:** 1 Key Laboratory of Grain Information Processing & Control, Ministry of Education, Henan University of Technology, Zhengzhou 450001, China; gehongyi2004@163.com (H.G.); gcy20001104@163.com (C.G.); zy_haut@163.com (Y.Z.); zwhjy2022@163.com (W.Z.); wh843754374@163.com (H.W.); 2 Henan Provincial Key Laboratory of Grain Photoelectric Detection and Control, Zhengzhou 450001, China; 3 College of Information Science and Engineering, Henan University of Technology, Zhengzhou 450001, China; 4 School of Artificial Intelligence and Big Data, Henan University of Technology, Zhengzhou 450001, China

**Keywords:** terahertz images, image classification, MobileViT-E, broken grains, deformed grains

## Abstract

The variety and content of high-quality proteins in sunflower seeds are higher than those in other cereals. However, sunflower seeds can suffer from abnormalities, such as breakage and deformity, during planting and harvesting, which hinder the development of the sunflower seed industry. Traditional methods such as manual sensory and machine sorting are highly subjective and cannot detect the internal characteristics of sunflower seeds. The development of spectral imaging technology has facilitated the application of terahertz waves in the quality inspection of sunflower seeds, owing to its advantages of non-destructive penetration and fast imaging. This paper proposes a novel terahertz image classification model, MobileViT-E, which is trained and validated on a self-constructed dataset of sunflower seeds. The results show that the overall recognition accuracy of the proposed model can reach 96.30%, which is 4.85%, 3%, 7.84% and 1.86% higher than those of the ResNet-50, EfficientNeT, MobileOne and MobileViT models, respectively. At the same time, the performance indices such as the recognition accuracy, the recall and the F1-score values are also effectively improved. Therefore, the MobileViT-E model proposed in this study can improve the classification and identification of normal, damaged and deformed sunflower seeds, and provide technical support for the non-destructive detection of sunflower seed quality.

## 1. Introduction

China is the world’s fourth-largest producer of sunflower seeds with the world’s sixth-largest planting area [1]. Sunflower seeds are one of the important oil crops and their seed kernels are rich in high-quality protein, vitamins, unsaturated potassium and phosphorus, with a fat content of 30–45%, and sometimes even more than 60%. The research and development of new protein foods have shown that the types and content of high-quality protein in sunflower seeds are higher than those of other cereals, and this protein can provide one of the most important sources of plant protein needed by the human body. However, sunflower seeds are prone to abnormalities such as breakage and deformities during planting, harvesting, and storage, which can be attributed to environmental factors and pest infestations. Therefore, there is an urgent need to establish a rapid, accurate, and non-destructive testing method to identify the quality of sunflower seeds.

The existing traditional inspection methods are mainly manual sensory evaluation methods [2], machine sorting [3] and X-ray [4]. However, manual inspection is subjective and prone to visual fatigue, misdetection and omission, affecting inspection efficiency. The machine sorting technology can detect the external characteristics of sunflower seeds, but it cannot detect their internal characteristics. X-ray methods can detect the internal quality of sunflower seeds, but they require professional operating skills and may cause radiation hazards to the operator due to the high energy. Therefore, research on the rapid, accurate, and non-destructive detection of sunflower seed quality is very important for achieving good sunflower seed quality and safety monitoring, as well as the healthy development of the sunflower seed industry.

Terahertz (THz) waves are located between the microwave and infrared light frequencies, in the band of 0.1–10 THz [5]. They have unique spectral properties, including low energy, non-contact detection, strong ability to penetrate a variety of media materials, etc. They can be used to detect internal defects in different objects, and at the same time, they have a strong communication transmission capability [6,7]. These properties have made it possible to apply THz technology in the fields of agricultural product safety testing [8], drug and biomedicine [9], grain storage quality testing, and communications [10]. It enables the non-destructive detection and diagnosis of biomolecules, as well as qualitative and quantitative analyses of the composition of substances. THz spectroscopy provides information about the physical, chemical and molecular structure of the target sample, and is suitable for the study and characterisation of fingerprint properties of substances. THz imaging is based on THz spectroscopy, where each pixel point of the image obtained by scanning and imaging the target sample represents a piece of spectral information [11]. The THz image of the sample is generated by simple data processing and analysis of the transmitted and reflected signals of the sample to be tested.

In recent years, THz waves have facilitated significant breakthroughs in the quality inspection of sunflower seeds, thanks to their advantages of non-destructive penetration and fast imaging. Sun et al. [12] selected insect-eaten, defective and intact sunflower seeds as samples and obtained their THz images. The authors modelled and predicted the fullness of these seeds with a coefficient of determination and root mean square error of prediction (RMSEP) of 0.91 and 4%, respectively. Lei et al. [13] developed a dual autoencoder (AE)–generative adversarial net (GAN) spectral dehulling semi-supervised model and, for the first time, evaluated the distribution of energy and moisture inside sunflower seed shells using non-destructive THz time-domain imaging systems. Yuan et al. [14] dealt with the problem of sample imbalance within the spectral information of sunflower seed imperfections collected by the THz time-domain spectral detection technique. The authors expanded the dataset of imperfections using the SMOTE algorithm, and achieved an improved detection accuracy of 92.59% using the recognition model constructed by the least-squares support vector-based recognition model mechanism. Lei et al. [15] acquired sunflower seed projections at different angles on a rotating stage by a custom-made THz time-domain imaging system, and reconstructed three-dimensional (3D) sunflower seed images using the inverse Randon transform. The two-dimensional (2D) and 3D fullness of sunflower seeds was calculated from the volume and area ratios of the whole seeds to seeds. However, as the THz imaging technique is affected by the system and environment noise during image acquisition, it may suffer from problems such as low resolution, unclear key image information and vague edge information, which affect the final sunflower seed detection and identification results.

In order to detect the sunflower seed quality more effectively, this paper introduces the efficient multi-scale attention (EMA) mechanism as a lightweight network, which ensures a high detection accuracy and eliminates the interference of redundant information. At the same time, the MV2 structure is improved, and the exponential linear unit (ELU) activation function is used to accelerate the convergence speed of the network. A non-destructive sunflower seed quality detection model known as MobileViT-E is proposed, which improves the detection accuracy by obtaining detailed features with the ability to discriminate subtle differences.

## 2. Materials and Methods

### 2.1. Experimental Equipment and Principles

**Figure 1:**
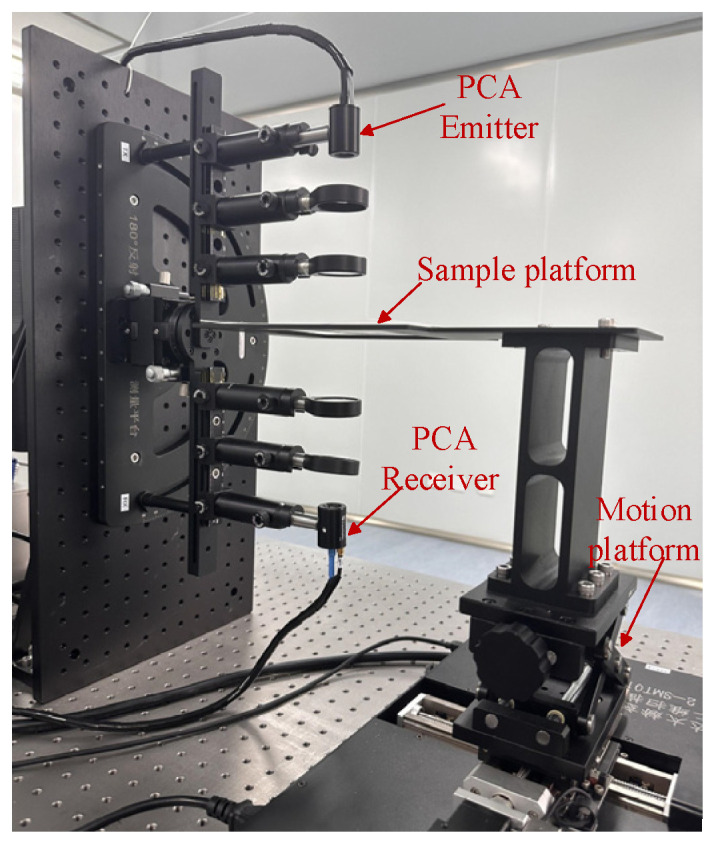
Photograph of the QT−TO1000 terahertz spectral imaging setup.

**Figure 2:**
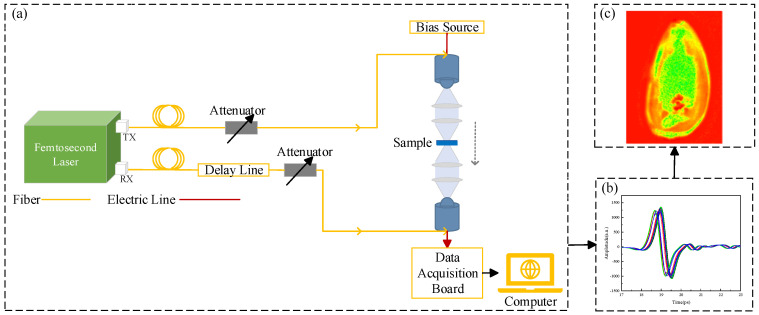
Schematic diagram of QT−TO1000 terahertz spectral imaging. (**a**) Basic structure of the system, (**b**) THz spectrum of the sample, (**c**) THz image of the sample.

The experimental equipment is a commercial THz 3D tomographic system, the QT−TO1000 from Qingdao Quenda THz Technology Ltd. in Qingdao, China, as shown in Figure 1. In this experiment, the transmission mode was used for sample detection. The THz frequency was 0.1−3 THz, and the spectral dynamic range was greater than 60 dB. The schematic diagram is shown in Figure 2. Initially, the sample was placed on a 2D scanning platform, with the scanning time range set to 90 ps. The fibre-optic femtosecond laser sent out two femtosecond laser beams, one of which was used as a pumping light source. This beam was forwarded to the transmitting antenna through the optical fibre to generate a broadband THz wave under a certain bias voltage. Subsequently, it was irradiated onto the sunflower seed sample after being focused by the lens group and received by the THz receiving antenna. The second femtosecond laser beam acted as a probe light source and was transmitted to the THz receiving antenna through an optical fibre and a time−delay device, which generated photogenerated carriers. A current signal was produced in the presence of THz waves. Finally, the detected signals were transmitted to the computer side via a data acquisition card for further processing and analysis.

### 2.2. Sample Preparation and THz Image Data Acquisition

**Figure 3:**
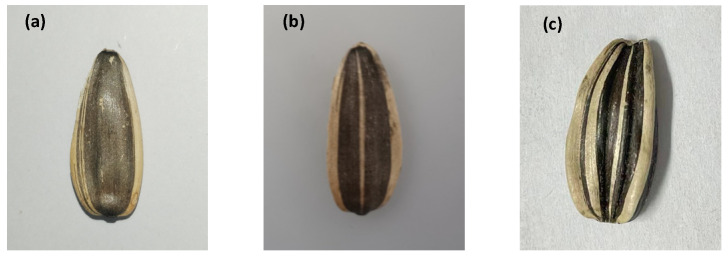
Camera images of sunflower seeds. (**a**) Normal grain, (**b**) broken grain, (**c**) deformed grain.

The sunflower seeds used in this paper were purchased from Hangjin Houqi, Bayannur City, Inner Mongolia Autonomous Region, China. They included seven different varieties of sunflower seeds: 363, 361, 601, 2399, Lefeng 3.0, Tongqing No. 5 and Tongqing No. 6. Deformed grains of sunflower seeds were randomly selected from the samples. Some normal samples were selected to prepare different internally broken sunflower seeds for THz imaging detection. Figure 3 shows the images of normal, deformed and broken sunflower seeds. The samples were placed on a 2D scanning platform for THz transmission imaging. The maximum scanning area of the system was 100×100 mm, the time range of scanning was chosen as 90 ps, and the spatial resolution was set to 0.1 mm. The acquired image data were stored in 3D form, containing the spectral and spatial information of sunflower seed samples.

**Figure 4:**
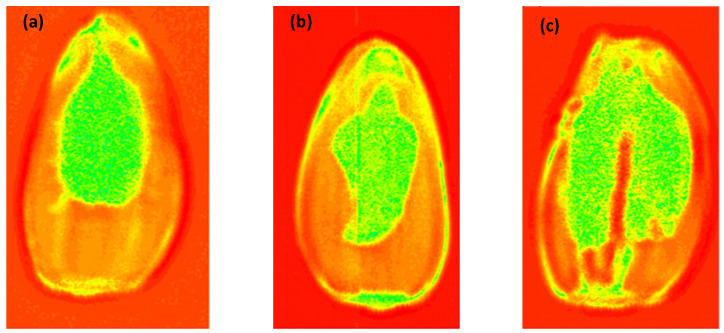
THz images of sunflower seeds in transmittance mode. (**a**) Normal grain, (**b**) broken grain, (**c**) deformed grain.

Figure 4 shows that the contour features of sunflower seeds can be clearly observed after peak-to-peak imaging. Due to the strong penetration ability of THz, the internal characteristic information of the three kinds of sunflower seeds was significantly contrasted, and the damaged positions were clearly visible. The absorption intensity of THz waves was different for various materials. Since the seed kernel was thicker than the outer shell, the image characteristics of deformed grains could also be clearly observed. Because of the limitation of the detection platform, the THz image information of each sunflower seed sample was acquired individually.

### 2.3. Proposed Work

#### 2.3.1. Proposed MobileViT-E Network Architecture

**Figure 5:**
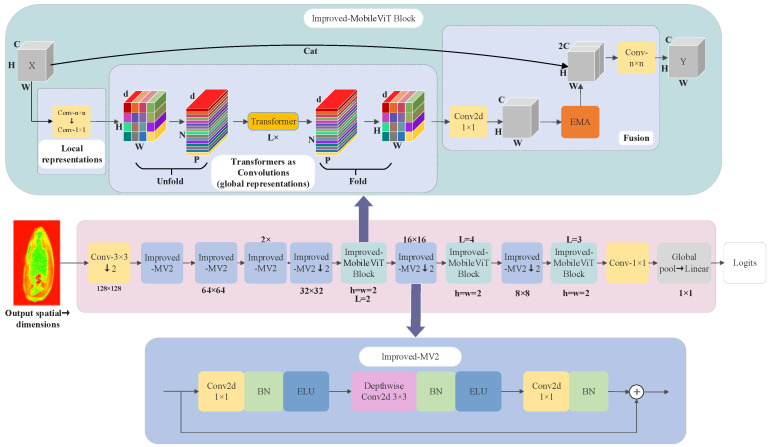
MobileViT-E network architecture diagram.

This study proposed a sunflower seed detection model to achieve accurate and non-destructive detection of sunflower seed quality, which was named the MobileViT-E network. First, MobileViT [16], a lightweight network with a small number of parameters, was chosen as the base network. The redundant features of the image were eliminated by reducing the information interference and focusing on the basic detailed features of the image. An EMA [17] mechanism was introduced into the MobileViT block, which reduced the computational cost of the model while simultaneously preserving the information of each channel. It decreased the training time of the model, and improved the detection accuracy by processing the features in parallel at different scales. Meanwhile, in order to solve the vanishing gradient and exploding gradient problems, the activation function in the MV2 structure in the MobileViT network model was improved to speed up the convergence of the network and improve the performance of the model. Figure 5 shows the model network structure, which is mainly composed of ordinary convolution, Improved MobileViT block, Improved MV2 structure, global pooling and fully connected layer. The blocks to be downsampled were marked by “↓”. 

A THz image of sunflower seeds with a size of 256×256 was input into the MobileVIT-E model. Initially, the input image was downsampled twice by 3×3 standard convolution in order to extract preliminary features. Subsequently, four Improved MV2 blocks were employed for two additional downsamplings to further extract image features. The ELU activation function improved the learning speed and classification accuracy of the proposed network. Then, the feature maps were input into the Improved MobileViT blocks. Within these blocks, local convolution captured spatial information, while Transformer coding was used to understand global relationships. The EMA mechanism adjusted specific batch dimensions, which improved the proposed model performance and processing speed. Finally, local and global features were fused to generate the final output. The processed feature map was alternately input into the Improved MV2 blocks and Improved MobileViT blocks, which produced an 8×8 tensor. The tensor underwent 1×1 convolution for channel compression. It was classified using a linear classifier after global average pooling to produce the final classification result of sunflower seed images.

#### 2.3.2. MobileViT Network Architecture

MobileViT [16] is a lightweight hybrid architecture model combining the convolutional neural network and a Transformer. Traditional visual Transformer models have a large number of parameters, which makes them computationally inefficient. In contrast, the MobileViT model aims to achieve lightweight but high-performance classification performance by reducing the number of parameters and computational complexity of the model. The main components of the model are the MV2 inverted residual module and MobileViT block cascade.

**Figure 6:**
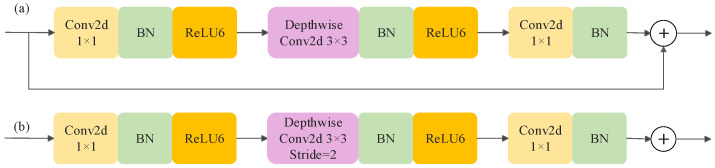
Structure of the MV2 network. (**a**) Stride = 1; (**b**) stride = 2.

The structure of the MV2 module is shown in Figure 6. It is based on depth-separable convolution, inverted residual connection and linear bottleneck to effectively achieve local feature extraction while reducing the number of model parameters. The inverted residual structure improves the feature dimensions using 1×1 point-by-point convolution to increase the feature granularity, and subsequently uses 3×3 depth convolution to achieve feature extraction from each channel, thus reducing the number of model parameters. The features are then downsampled by 1×1 point-by-point convolution to reduce the output dimension of the model. The residual connection is only used under specific conditions: feature loss. When the step size is 1 and the input and output dimensions are the same, the introduction of residual structure can be avoided. When the step size is 2, the series connection is used to downsample the feature layer.

**Figure 7:**
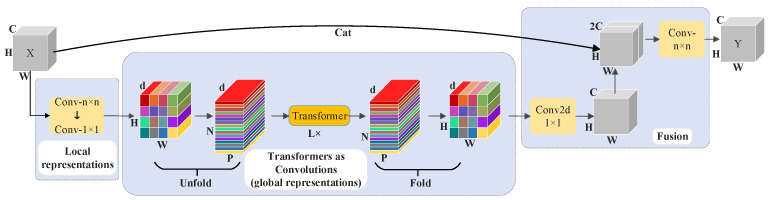
MobileViT block structure.

The structure of the MobileViT block is shown in Figure 7, and it is divided into three parts: local feature extraction, global feature extraction and feature fusion. The input of the block is the feature map $X\in R^{C\times H\times W}$ whose height, width and number of channels are denoted by H, W and C, respectively. First, local spatial information in the map is acquired by using the standard convolution of kernel size n×n. Second, the number of channels is adjusted by a convolution kernel of size 1×1, and a local feature map of size $X_{L}\in R^{H\times W\times d}$ is output to learn richer semantic information. Third, in order to learn the global representation with spatial induction bias, the feature map is divided into N non-overlapping image blocks $X_{J}\in R^{P\times N\times d}$ of size h×w, where P=h×w, N=H×W/P, and h and w are the height and width of the image blocks, respectively. Fourth, the relationship between the image blocks is then encoded using the L-group Transformer module to obtain the global semantic information, i.e., each pixel point after the Transformer operation contains the feature information from all pixel points. Fifth, the P×N×d sequence is reduced to the 1×1 pre-encoding dimension to obtain the H×W×d feature map. Last, a convolutional map of size is used to output a map with dimension C and combined in series with X to generate a new feature map of dimension 2C. The global and local features are fused using the fusion module to obtain the final output Y.

#### 2.3.3. EMA Mechanism

**Figure 8:**
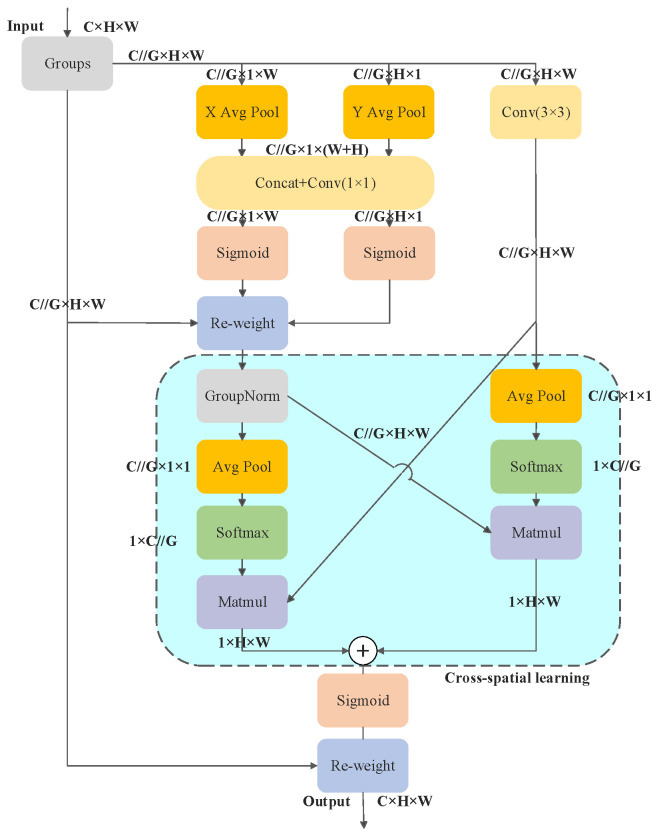
EMA module structure.

The efficient multi-scale attention (EMA) [17] mechanism achieves comprehensive feature aggregation by using cross-space learning to aggregate information from different spatial dimensions. It reconstructs some channels into batch dimensions and groups the channel dimensions into multiple sub-features to distribute the spatial semantic features uniformly within each group. The inter-channel dependencies are captured by modelling the information interactions across the channels, which improves the performance and processing speed of the model. Figure 8 shows the structure of the EMA, which consisted of three parts: feature grouping, parallel subnet and cross-space learning.

The EMA obtains diverse semantic information by dividing a given input feature map $X\in R^{C\times H\times W}$ into G sub-feature maps along the channel dimension. These groups can be written as $X=\left[X_{1},X_{2},\cdots X_{G-1}\right]$, $X_{I}\in R^{C//G\times H\times W}$. The attention channel weights for grouping the feature maps are extracted using three parallel branches, two of which are 1×1 branches and the remaining one is a 3×3 branch. In the 1×1 branch, the channel is encoded from two spatial directions using two one-dimensional (1D) global average pooling operations. In the 1×1 branch, the channel is encoded from two spatial directions using two 1D global average pooling operations. Subsequently, the two coded features are spliced along the vertical direction of the image so that they share the same 1×1 convolution operation without a dimensionality reduction in the 1×1 branch. Once the output of the 1×1 convolution is decomposed into two vectors, the 2D binomial distribution based on linear convolution is modelled using two nonlinear sigmoid functions. Finally, the two channel attention maps within each group are aggregated using simple multiplication to obtain diverse cross-channel interaction features between two parallel paths in the 1×1 branch. A 3×3 convolutional kernel is utilised in the 3×3 branch to capture multi-scale feature representations. Thus, the EMA not only encodes inter-channel information to adjust the importance of various channels, but also preserves the precise intra-channel spatial structural details.

In addition, the EMA implements a cross-space information aggregation strategy. The global spatial information is extracted from the 1×1 branch output using 2D global average pooling operation, as shown in (1):

(1) $$Z_{C}=\frac{1}{H\times W}\sum_{j}^{H}\sum_{i}^{W}x_{c}\left(i,j\right)$$

Subsequently, the nonlinear function softmax is used for 2D Gaussian mapping to fit the linear transformation and improve the computational efficiency. The output of the 3×3 branch is adjusted to align with the corresponding dimensional structure. At the same time, a dot product operation is performed using the results of the parallel processing stage to obtain the initial spatial attention map. The global spatial information in the 3×3 branch undergoes 2D global average pooling, and the output of the 1×1 branch is adjusted to match the corresponding dimensional structure and obtain the second spatial attention map, which retains the accurate spatial position information. Finally, the two spatial attention weight values are used to reweight and aggregate the inputs in a reweighting operation.

#### 2.3.4. *ELU* Activation Function

The nonlinear exponential linear unit (*ELU*) [18] activation function is used to introduce nonlinear properties in neural networks. It is mathematically formulated as follows:

(2) $$ELU(x)=\{\begin{array}{c} x,\;x>0\\ \alpha (\exp(x)-1),\;x\leq 0 \end{array}$$

The *ELU* activation function has a stronger learning ability and a faster training speed compared with other linear unsaturated activation functions. It exhibits a significant advantage in dealing with negative values, and thus it can effectively avoid the “dead neuron” problem of the *ReLU* function, which improves the learning speed and classification accuracy of the neural network. In addition, it can make the normal gradient resemble the unit natural gradient by reducing the effect of bias offset, which in turn accelerates the mean-to-zero learning process. Furthermore, when the input features are negative, the output activation values tend to saturate. This saturation helps to effectively reduce the changes in the next layer and the interference in the information transfer during backpropagation, thus enhancing the model’s robustness against noise. These properties make the *ELU* an efficient and reliable choice of activation function in deep learning.

### 2.4. Evaluation Metrics

In this study, *accuracy*, *precision*, *recall* and *F1-score* were used as evaluation metrics to assess the performance of the sunflower THz image detection model. *Accuracy* indicates the proportion of correct results out of the total results identified by the model. The *precision* measures the model’s ability to accurately judge positive instances, and the *recall* reflects the model’s ability to recall positive instances. The weighted summed average of *precision* and *recall* is represented by the *F1-score*. The higher values of these indicators show higher effectiveness of the model. The *accuracy* can be calculated using the following formula:

(3) $$accuracy=\frac{TP+TN}{TP+FN+FP+TN}$$

The formula for the *precision* is

(4) $$precision=\frac{TP}{TP+FP}$$

*Recall* can be calculated as

(5) $$recall=\frac{TP}{TP+FN}$$

The *F1-score* can be obtained using the following formula:

(6) $$F1-score=\frac{2\times precision\times recall}{precision+recall}$$

where TP denotes the number of correctly predicted positive cases, FP denotes the number of incorrectly predicted positive cases, FN denotes the number of incorrectly predicted negative cases, and TN denotes the number of correctly predicted negative cases.

## 3. Results and Discussion

### 3.1. Model Training

In this experiment, the dataset consisted of THz images of normal, broken and deformed sunflower seeds in different frequency domains. The whole dataset contained 2340 THz transmission images of sunflower seeds, which were divided into training and test sets in the ratio of 8:2. The training batch was set to 32, the training period was 60 epochs, and the input image size was 256 × 256 pixels. To ensure the objectivity of model performance comparison, the AdamW optimizer was used in all models, the initial learning rate was set to 5 × 10^−4^, and the cross-entropy was used as the loss function. The classification models were constructed and trained using the Pytorch deep learning framework. The hardware used for model training and testing consisted of an Intel (R) Core (TM) i5-13400F (2.50 GHz) processor with Windows 11 operating system and an NVIDIA GeForce RTX 3060 graphics card. In terms of software configuration, Python version 3.8 and CUDA 11.8 framework were utilised to accelerate the computations. The training samples and their corresponding labels were used as inputs to the network for training and testing the image classification task, respectively.

### 3.2. Comparison Experiments

In order to further demonstrate the superiority of the MobileViT-E model for sunflower seed quality detection, several classical deep learning network models, namely ResNeT-50, EfficientNeT, MobileOne and MobileViT, were compared with the MobileViT-E model in this study. Table 1 shows the training results: the MobileViT-E model demonstrated significant advantages in the classification of sunflower seeds. It achieved the highest accuracy, precision, recall and F1-score values of 96.30%, 96.35%, 96.30% and 96.31%, respectively. In comparison with the ResNet-50, EfficientNet, MobileOne and MobileViT models, the accuracy of the proposed model was improved by 4.85%, 3%, 7.84% and 1.86%, respectively. These results indicated that the proposed model was more capable of processing complex image features and could accurately identify nuances and details in sunflower seed features.

In comparison, the MobileViT model achieved the second-highest classification performance, with accuracy, precision, recall and F1-score values of 94.44%, 94.64%, 94.44% and 94.49%, respectively. The results of the EfficientNet model were similar to those of the MobileViT model, with accuracy, precision, recall and F1-score values of 93.3%, 94.1%, 93.3% and 93.2%, respectively. However, the MobileOne model exhibited relatively lower performance, with accuracy, precision, recall and F1-score values of 88.46%, 89.08%, 88.46% and 88.22%, respectively. These results might indicate the limitations in its image feature extraction.

**Figure 9:**
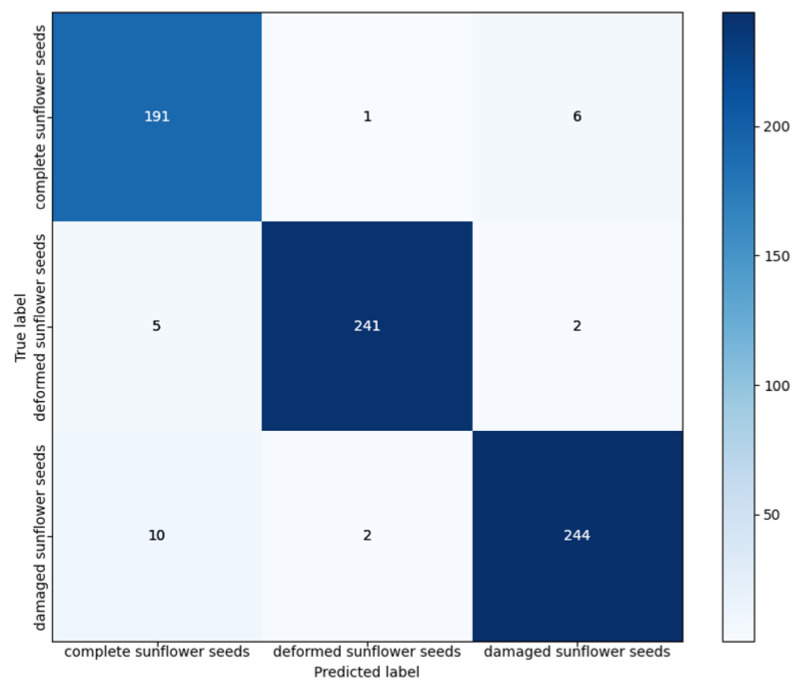
Confusion matrix of the MobileViT-E model on the sunflower seed dataset.

Meanwhile, to show the prediction performance of the MobileViT-E model more clearly for different categories of sunflower seeds, the model with the highest accuracy on the test set was selected, and its confusion matrix is plotted in Figure 9. It could be observed that the proposed model had the best prediction performance on the deformed grains, with an accuracy of 97.17%. Five samples were incorrectly predicted as normal grains, and two samples were incorrectly predicted as broken grains. The second-best prediction was on normal sunflower seeds, with an accuracy of 96.46%. In this case, one sample was incorrectly predicted as deformed grains, and six samples were incorrectly predicted as broken grains. Finally, the prediction performance for broken grains was the worst, with an accuracy of only 95.31%. There were 12 errors, with 10 samples predicted as normal grains and two samples predicted as deformed grains. This might be caused by the presence of noise and other disturbances in the images, which led to unclear features in some of the images and affected the classification performance of the model. Overall, the MobileViT-E model utilised the Transformer architecture to effectively capture global and local features when processing the sunflower seed images, achieving accurate classification of normal, broken and deformed grains.

### 3.3. Ablation Experiments

A series of ablation experiments were carried out to compare the performance differences under different model configurations. The actual contribution of each module to the model performance and the performance enhancement of the improved model compared to the baseline model MobileViT, as well as the MobileViT + EMA and MobileViT + ELU models, were verified. Table 2 shows the performance comparison, where “√” indicates that the module was used in this network, and “×” indicates that the module was not used.

Table 2 shows that the inclusion of EMA and ELU modules in this study substantially improved the classification recognition accuracy, with the prediction accuracy increased from 94.44% to 96.30% in the baseline model, and the precision, recall and F1-score also significantly improved by 1.71%, 1.86% and 1.82%, respectively. When only the ELU module was used, the accuracy, precision, recall and F1-score values of the model showed minor improvements of 0.15%, 0.16%, 0.15% and 0.05%, respectively. When only the EMA module was introduced, the accuracy of the model was improved by 1.29% compared to the baseline model, and the precision, recall and F1-score values were also improved by 1.12%, 1.29% and 1.22%, respectively. Overall, these improvements were significant compared to the baseline model, but they were still lower than those exhibited by the MobileViT-E model. These results showed that the MobileViT-E model was the most effective in classifying normal, broken and deformed grains of sunflower seeds, and could achieve accurate and non-destructive detection of sunflower seed quality.

In this paper, the proposed MobileViT-E model combined the feature extraction capabilities of deep learning with the self-attention mechanism of Transformers, which demonstrated significant advantages in sunflower seed quality detection. Compared to four other image classification models, including ResNet-50, EfficientNet, MobileOne and MobileViT, the proposed model achieved higher accuracy in detecting normal, damaged and deformed sunflower seeds in THz images. Additionally, the introduction of the EMA mechanism and ELU activation function was further verified by ablation experiments to effectively improve the detection capability. However, the samples were affected by the internal noise of the system during the imaging process. This interference reduced the image details and caused the loss of image edge information. Although the model achieved a recognition accuracy of 96.30% on the sunflower seed dataset, its detection precision still required improvement. This was especially evident for damaged seeds, with an accuracy of only 95.31%. Therefore, future improvements should focus on the construction of network models with higher detection accuracy. Meanwhile, the data used in this study were limited to broken and deformed grains of sunflower seeds. In the future, the type and number of sunflower seed samples can be expanded to construct a wider dataset, and the algorithm can be applied to the detection of other agricultural features to improve the generalisation of the model.

## 4. Conclusions

In this study, we proposed a non-destructive inspection model MobileViT-E for sunflower seed quality. It was based on the MobileViT model and used the Transformer architecture to extract the multi-scale features of sunflower seed images. It acquired and analysed the subtle features of the images through the self-attention mechanism and global feature extraction. The EMA mechanism was introduced in the MobileViT block to further improve its performance. This optimised the model’s attention on the basic image features and reduced the interference from irrelevant information while retaining the necessary information from each channel to reduce the computational cost. Additionally, the ELU activation function was used in the MV2 structure to avoid the vanishing gradient problem, speed up network training, and improve the model’s generalisation ability. The experimental results showed that the MobileViT-E model improved the recognition accuracy by 4.85%, 3%, 7.84% and 1.86% compared with the ResNeT-50, EfficientNeT, MobileOne and MobileViT models, respectively. Thus, the proposed model could significantly improve the sunflower seed quality detection accuracy. However, the current study was mainly limited to the laboratory environment, and there is a need to further verify the model’s stability and generalisation ability in real complex environments. In the future, the MobileViT-E model can be improved and optimised in various ways. For example, a pre-trained model can be used for transfer learning to accelerate the model convergence and improve the detection accuracy. In addition, a richer and more extensive sample database can be constructed to achieve an efficient and accurate quantitative and qualitative analysis of the sunflower seed quality. The proposed model will not only enhance the standardisation of agricultural product safety testing, but also contribute to the application of machine learning and other advanced analytical techniques in the field of food quality control.

## Figures and Tables

**Table 1:** Recognition performance of deep learning networks on THz images of sunflower seeds.

Model	Accuracy/%	Precision/%	Recall/%	F1-Score/%
ResNeT-50 [19]	91.45	92.00	90.53	90.93
EfficientNeT [20]	93.30	94.10	93.30	93.20
MobileOne [21]	88.46	89.08	88.46	88.22
MobileViT [16]	94.44	94.64	94.44	94.49
MobileViT-E	96.30	96.35	96.30	96.31

**Table 2:** Performance comparison of different modules in sunflower seed image classification model.

Model	MobileViT	EMA	ELU	Accuracy/%	Precision/%	Recall/%	F1-Score/%
1	√	×	×	94.44	94.64	94.44	94.49
2	√	√	×	95.73	95.76	95.73	95.71
3	√	×	√	94.59	94.80	94.59	94.54
4	√	√	√	96.30	96.35	96.30	96.31

## Data Availability

The data presented in this study are available on request from the corresponding author. The data are not publicly available due to privacy restrictions.
